# The Liverpool Policy and the Voluntary System

**Published:** 1913-06-14

**Authors:** 


### MUNICIPAL AND COUNTY COUNCIL GRANTS FOR VOLUNTARY HOSPITAL CONSTRUCTION.

### The Liverpool Policy and the Voluntary System.

An unobtrusive notice in the press concerning powers granted recently to the Liverpool Corporation by the Local Legislation Committee of the House of Commons discloses a new and advanced policy in hospital construction at Liverpool. By these new powers £5,000 may be contributed from Corporation funds to the Royal Infirmary for rebuilding the out-patient department; £7,500 for a rate-supported Accident Hospital at Garston; and £1,750 towards the rebuilding of St. Paul's Eye Hospital. It will be noted that these grants will be, when made, for structural outlay. Power is given under the Act to grant such further sums as may be necessary. Manchester for many years, as we have often pointed out, has occasionally made grants to the Royal Infirmary towards the expenses of its site and buildings. It will be noted in the case of Liverpool that two of the grants are to be made to the building fund of two voluntary hospitals—*i.e.*, the Royal Infirmary and the St. Paul's Eye Hospital. The Accident Hospital at Garston, near Liverpool, is, and always has been, supported by local rates and no further information is obtainable about it. It is evident, however, that to make a grant to such an accident hospital out of the rates has no feature of novelty.

### The Pro Rata System.

In dealing with the changes in regard to hospital matters which must ultimately result from the coming into force of the National Insurance Act we have consistently advocated a policy whereby, by the adoption of the *pro rata* system, the voluntary principle of hospital support and government may be maintained, and provision made for an adequate increase in existing hospital accommodation to enable all insured persons needing hospital care to be provided with it by agreement, through the Insurance Commissioners, with the hospital authorities and the sanction of the Local Government Board. This plan would involve the granting of loans, with the sanction of this Board, to the voluntary hospitals to defray the cost of buildings on the basis of a loan at interest redeemable in a fixed number of years by means of a sinking fund. The interest and sinking fund (4 per cent. in all) to be a first charge upon the payments received to recoup the *pro rata* cost of maintenance and treatment of the patients in the new buildings thus provided. Recent discussions and the views expressed by leaders of public opinion throughout the country make it clear that the people desire the maintenance of our present system of voluntary hospitals. Grants made by municipalities and County Councils to voluntary hospitals for building purposes on the lines followed first in Manchester and now at Liverpool should, therefore, help and not hinder the perpetuation of the voluntary system.

We are interested to know, from a report in the *Manchester Evening News* of the 29th ultimo, of interviews a member of their staff had with the Lord Mayor, Sir Charles Behrens (the Deputy Lord Mayor), and the Chairman and Secretary of the Manchester Royal Infirmary Board, that the two former gentlemen do not appear to realise that in making a grant for building purposes to the Liverpool Royal Infirmary, Liverpool is but following a course taken occasionally in the past by the Manchester Corporation. At the present time Manchester gives a subscription of £2,000 a year to the various hospitals, which is divided amongst them in certain proportions. The Manchester Corporation also contributes under the National Insurance Act to the Manchester Consumption Hospital.

### The Position of Hospitals and Ratepayers.

We are glad to note in this connection that Sir Charles Behrens, whilst generally favouring more liberal assistance to the local voluntary hospitals through the Corporation, properly insisted that the Manchester Council should exercise infinite care in regard to this matter, with a view to prevent a large increase in the present high rates. We dwell upon this point because fear has been expressed that an increase in rates may become general owing to certain remarks made by Mr. Middlebrook, a Liberal M.P. for Yorkshire, as chairman of the House of Commons Local Legislation Committee, to the effect that he hoped the experiment which the Liverpool Corporation were empowered to make would prove to be an example to the whole country. We hold strongly to the principle that municipal authorities should everywhere be prepared, when invited by the voluntary hospital managers to do so, to make liberal contributions to any building and equipment fund raised to extend the accommodation of local voluntary hospitals where it is clearly shown that the requirements of the population justify and even compel such additional provision. At the same time the point raised by Sir Charles Behrens must everywhere be kept in mind, and it should exercise a wholesome check upon any attempt to interfere with the present system of administration of voluntary hospitals by imposing large contributions from municipal funds upon some or all of these institutions.

It is interesting in this connection to remember that the County Councils and other local authorities have tried to impose a new and heavy charge upon the voluntary hospitals by inducing them for very inadequate payments to undertake the grave responsibility and heavy cost of treating all the special cases sent from public rate-supported schools to the voluntary hospitals. In no single instance that we have been able to discover has this attempt proved in practice either desirable or successful. The hospital requirements for rate-supported school children should be met by the establishment of school clinics; but we regret to know that the school clinics of the County Council in London, for instance, as at present conducted, are not a credit to the County Council, nor do they invariably supply either the skilled advice or the perfected organisation which the parents of the children have a right to demand at the hands of the local authorities. The present condition of some of these school clinics, as they have been described to us, is a disgrace to the London County Council, and it is time that that body appointed an independent commission to investigate the present condition of these clinics, tuberculin dispensaries, &c., and to bring about the necessary reforms. All the existing evils, as we understand it, may be traced to the influence of party municipal government. We are assured that there are at least two notable examples in London of members of the profession of high qualifications and full knowledge who, by their outspokenness, have rendered themselves unpopular with political county councillors and have so been treated in a manner which entails grave expense and disadvantage upon the ratepayers with grave injustice and other evils to the practitioners concerned.

The *Manchester Evening News* also interviewed Sir William Cobbett and the Secretary of the Manchester Royal Infirmary. Sir William Cobbett said that he could not express any view on the question of the desirability or otherwise of the Liverpool policy; he had not considered the matter and had no views upon it. Mr. Carnt was also unable to express a decided opinion, and he wanted to know what power the Liverpool Corporation may desire to assume over the voluntary hospitals who accept grants on account of buildings.

We shall be glad to receive the views of other hospital managers throughout the country. It is plain that the Liverpool and other Corporations and local authorities which may make grants for building purposes to a voluntary hospital should not attempt in any way to interfere with, or be represented upon, the management of such an institution. Such authorities may reasonably be contented to have the right to inspect such buildings as may be erected by money supplied by them under suitable conditions to be mutually agreed between the hospital authorities and the representatives of each authority adopting such a policy.

